# Exposure of pregnant women and their children to pyrethroid insecticides in Rio de Janeiro, Brazil

**DOI:** 10.3389/fpubh.2023.1274724

**Published:** 2023-12-14

**Authors:** Amanda Friaes Martins, Aline Souza Espindola Santos, Josino Costa Moreira, Volney de Magalhaes Câmara, Carmen Ildes Rodrigues Froes Asmus, Ana Cristina Simoes Rosa, Paolo Vineis, Armando Meyer

**Affiliations:** ^1^Public Health Program, Public Health Institute, Federal University of Rio de Janeiro, Rio de Janeiro, Brazil; ^2^Occupational and Environmental Health Branch, Public Health Institute, Federal University of Rio de Janeiro, Rio de Janeiro, Brazil; ^3^School of Medicine, Maternity School Hospital, Federal University of Rio de Janeiro, Rio de Janeiro, Brazil; ^4^Center for Studies of Human Ecology and Worker's Health, National School of Public Health, Oswaldo Cruz Foundation, Rio de Janeiro, Brazil; ^5^MRC Centre for Environment and Health, School of Public Health, Imperial College London, London, United Kingdom

**Keywords:** pyrethroids, pregnant women, children, Rio de Janeiro, Brazil

## Abstract

**Background:**

Pyrethroids are commonly used insecticides in Brazil. Gestational and early childhood exposure to pyrethroids has been linked to adverse health effects, including neurodevelopmental delays, behavioral issues, and endocrine disruption. This study evaluated the exposure of pregnant women and their children to pyrethroid insecticides in Rio de Janeiro, Brazil.

**Methods:**

Creatinine-adjusted levels of the pyrethroid metabolites 3-phenoxy benzoic acid (3-PBA) and 4-fluoro-3-phenoxybenzyl acid (4-FPBA) were measured in the urine of 142 pregnant women and their children at birth and in the first, third, and 6th months of life.

**Results:**

The geometric mean (GM) and 95% confidence interval (95% CI) of 3-PBA and 4-FPBA urinary concentrations in pregnant women were 0.50 (0.37–0.67) and 0.37 (0.05–2.90) ng/mg, detected in 47.2 and 10.6%, respectively. Urinary concentrations of 3-PBA in the children were 0.18 (0.15–0.23) ng/mg at birth, 0.36 (0.08–1.56) ng/mg at 1-month-old, 0.68 (0.36–1.27) ng/mg at 3-month-old, and 1.36 (0.77–2.42) ng/mg at 6-month-old, and the detection rates were respectively 10.8, 9.4, 20.9, and 20.7%.

**Discussion:**

This study is one of the few that has evaluated the urinary concentrations of pyrethroids in newborns and children in their 1^st^ year of life. The results of this study show that children's exposure to pyrethroids significantly increases after birth.

## Introduction

Pyrethroids are a group of synthetic insecticides derived from natural pyrethrins found in the *Chrysanthemum cinerariaefolium* that show better environmental stability and performance (1, 2). Their use has been steadily increasing, and in 2021, the global pyrethroid market reached a size of 3.4 billion dollars (3). Insecticides are the third most used class of pesticides in Brazil (4). They are utilized in agricultural settings, and urban areas to control mosquitoes and other insects that transmit diseases such as dengue, chikungunya, and zika virus (5). These substances are also used as insect repellents and to treat ectoparasites in animals and humans (6, 7). In 2021, the Brazilian Association of Industries of Hygiene, Cleaning and Sanitizing Products for Domestic and Professional Use reported the sale of 1.26 million units of aerosol and 29,000 electrical devices containing pyrethroids in Brazil (8).

Household exposure to pyrethroids most commonly occurs through inhalation, ingestion, and, to a lesser extent, dermal contact due to their use as aerosols and topical formulations for treating pediculosis (9, 10). Studies have shown that pyrethroids are widespread in the general population (11, 12). These insecticides can cross the placenta and potentially harm the fetus's nervous and immune systems (13). Research has linked prenatal exposure to pyrethroids with impaired birth outcomes and child development (14, 15). For example, in North Carolina, USA, a case-control study concluded that residential proximity with the application of the pyrethroid cyhalothrin was associated with an increased chance of developing some congenital malformations, especially atrial sept defects (15), while in a birth cohort conducted in Xuanwei County, China, urinary levels of pyrethroid metabolites in the first and second gestational trimesters were associated with lower neurodevelopmental performance, assessed by the Bayley Scales of Infant and Toddler Development, in 1-year-old children (16). Several studies have shown an association between exposure to pyrethroids and an increased risk of language and neurobehavioral disorders, as well as neuromotor and neurocognitive effects in children (17–20). In the VHEMBE Cohort, South Africa, exposure to pyrethroids was associated with decrements in Social-Emotional scores of neurodevelopment at 1 year of age, and with decrements in Language Composite scores and Expressive Communication scores at the age of 2 (17). In children aged 6–9 years who lived near banana plantations in Costa Rica, urinary levels of 3-PBA were associated with poorer information processing speed capabilities (18). In France, Viel et al. concluded that exposure to pyrethroids negatively affected the neurobehavioral development of 6-year-old children enrolled in the PELAGIE mother-child cohort (19). Children aged 8–15 years old, who participated in the 2001–2002 National Health and Nutrition Examination Survey (NHANES) with urinary levels of 3-PBA above the limit of detection (LOD) were twice as likely to have Attention Deficit Hyperactivity Disorder compared with those below the LOD (20).

Despite its large use, limited studies in Brazil have evaluated pyrethroid concentrations in humans, especially in pregnant women and children. A study conducted in the Metropolitan area of Rio de Janeiro estimated that 87.5% of the 1,015 interviewed individuals had used household pesticides in the previous year and 81.6% of them mentioned aerosol spray-containing pyrethroids (21). Corcellas et al. detected pyrethroids in all breast milk samples (*n* = 17) collected from urban and rural populations in Brazil (22). In this study, we aimed to evaluate the exposure levels of pyrethroids in pregnant women and their children residing in the city of Rio de Janeiro, Brazil.

## Methods

### Participants and recruitment

This was a descriptive cross-sectional analysis of the data collected during the pilot phase of the Rio Birth Cohort (PIPA Study), a longitudinal study designed to investigate the role of gestational exposure to selected environmental pollutants on fetal and child development in the city of Rio de Janeiro, Brazil. Pregnant women in the third trimester and over 16 years old, who agreed to participate in the study were recruited between October and November of 2017 at the Federal University of Rio de Janeiro maternity hospital. This is a reference hospital for eight family health units responsible for the prenatal care of populations living in low-income communities in Rio de Janeiro city. The children of the enrolled mothers were monitored through clinical and laboratory tests from birth to 6 months. The study was approved by the Ethics Committee of the Maternity Hospital, Federal University of Rio de Janeiro (protocol 2.092.440). Each participant was informed of all relevant aspects of the study and voluntarily signed a consent form.

For the pilot study, we intended to enroll at least 10% of the pregnant women expected to participate in the main cohort study, calculated in 1,000 women. Every pregnant woman assigned to give birth at the Federal University of Rio de Janeiro maternity hospital, must visit the hospital between gestational weeks 27 and 28. During these visits, we randomly invited 209 eligible pregnant women, and 142 (68%) accepted the invitation to participate in the study. We collected information on their sociodemographic, lifestyle, and prenatal details using an enrollment questionnaire. From October 2017 to February 2018, a total of 135 deliveries took place at the maternity school, and we excluded four twin pairs from the study. We obtained birth, anthropometric, and clinical information on the children from the hospital records. We collected urine samples from all 142 pregnant women, 78 (58%) newborns, 53 (39%) 1-month-old, 67 (50%) 3-month-old, and 58 (43%) 6-month-old children.

### Laboratory analysis

We collected 40 ml of urine samples from all pregnant women and stored them in a refrigerator between 2°C and 7°C for a maximum of 48 h. We then transported the samples in isothermal boxes with recyclable ice to the lab, where they were stored in a freezer at −20°C until analysis. To analyze the metabolites 3-PBA and 4-FPBA, we used solid phase extraction and liquid chromatography coupled with triple quadrupole sequential mass spectrometry (23). All chromatographic procedures were conducted at the toxicology lab, at The National School of Public Health, Oswaldo Cruz Foundation, Brazil. We followed the standard DOQ-CGCRE-008, Revision 4, from the National Institute of Metrology, Quality, and Technology (24) to validate the analytical procedures. The method's ideal working range was between 0.5 and 15 ng mL^−1^, with good precision and accuracy. The limits of detection and quantification were 0.05 and 0.25 μg/L^−1^ for 4-FPBA, and 0.06 and 0.20 μg/L^−1^ for 3-PBA, respectively. We conducted analyte recovery tests at concentrations of 0.5, 5, and 15 μg/L^−1^ to verify the method's accuracy. The results we obtained were 95% for 3-PBA and 84% for 4-FPBA.

Urinary concentrations of the pyrethroid metabolites were corrected for creatinine levels, to avoid distortions caused by variations in urine dilutions (25). Results were expressed in nanograms of analyte per microgram of creatinine (ng mg^−1^). Urine creatinine was measured spectrophotometrically using Jaffe's reaction method.

### Statistical analysis

Descriptive analysis included the frequency of maternal characteristics such as age (16–19; 20–39; ≥40 years), ethnicity (white; non-white), family monthly income in Brazilian reais (≤ 1,733.00; 1,733.34–3,000.00; >3,000.00), schooling (High school or less; higher education), body mass index (<25.0; 25.0–29.9; ≥30.0), alcohol consumption (yes; no), smoking (yes; no), and passive smoke (yes; no). Children's characteristics included sex, birth weight (<2.500; ≥2.500), size for gestational age (small; appropriate; and large for gestational age), gestational age (<37 weeks; >37 weeks), and Apgar 5th min (≤ 7; >7). Only 3-PBA was found in the urine samples of newborns and 1, 3, or 6-month-old children. The GM and 95% CI were reported for urinary 3-PBA concentrations of pregnant women, newborns, and children aged 1, 3, or 6 months. We used the Kruskal-Wallis test, followed by *post hoc* analysis using Dunn's test, to compare the 3-PBA concentrations across the children's age groups. We used STATA V.18 and SPSS V.29 for all analyses.

## Results

Table 1 describes the main characteristics of the studied population. Most pregnant women (82.7%) were between 20 and 39 years old and non-white (73.6%). Approximately 45.2% reported an average household monthly income between R$ 1,733 and 3,000 (USD 306–530), but 31.3% reported it to be below R$ 1,733. Most of the participants (76.3%) had a high school degree or less. Regarding pre-gestational BMI, 47% of the mothers were normal weight, 33.3% were overweight, and 19.7% were obese. Alcohol consumption during pregnancy was reported by 46.6% of the women, while 10.9% reported smoking before pregnancy, and 9.3% smoked during pregnancy. In addition, 48.4% reported living with a person who smoked. Among the children, males were slightly more frequent (54.9%) than females. Seven percent of the children had low birth weights (<2,500 g), 7.8% had a small size for gestational age, 9.8% were delivered before the 37th week of gestation and 6.2% showed an Apgar score below 7.

**Table 1:** Main characteristics of the studied population.

	**N**	**%**
**Mothers**		
**Age (years)**		
16–19	14	10.5
20–39	110	82.7
≥40	9	6.8
**Ethnicity**		
White	34	26.4
Non-white	95	73.6
**Monthly income** ^*^		
≤ 1,733.33	36	31.3
1,733.34–3,000.00	52	45.2
>3,000.00	27	23.5
**Schooling**		
High school or less	100	76.3
Higher education	31	23.7
**Body mass index**		
< 25	55	47.0
25–29.9	39	33.3
≥30	23	19.7
**Alcohol consumption**		
Yes	61	46.6
No	70	53.4
**Smoking**		
Never	103	79.8
Before pregnancy	14	10.9
During pregnancy	12	9.3
**Passive smoking**		
Yes	62	48.4
No	66	51.6
**Children**		
**Sex**		
Male	62	54.9
Female	51	45.1
**Birth weight (g)**		
< 2.500	8	7.0
≥2.500	107	93.0
**Size for gestational age**		
Small for gestational age	8	7.8
Appropriate for gestational age	84	81.5
Large for gestational age	11	10.7
**Gestational age (weeks)**		
< 37	10	9.8
≥37	92	90.2
**Apgar 5th min**		
≤ 7	8	6.2
>7	121	93.8

^*^Brazilian reais.

**Figure 1:**
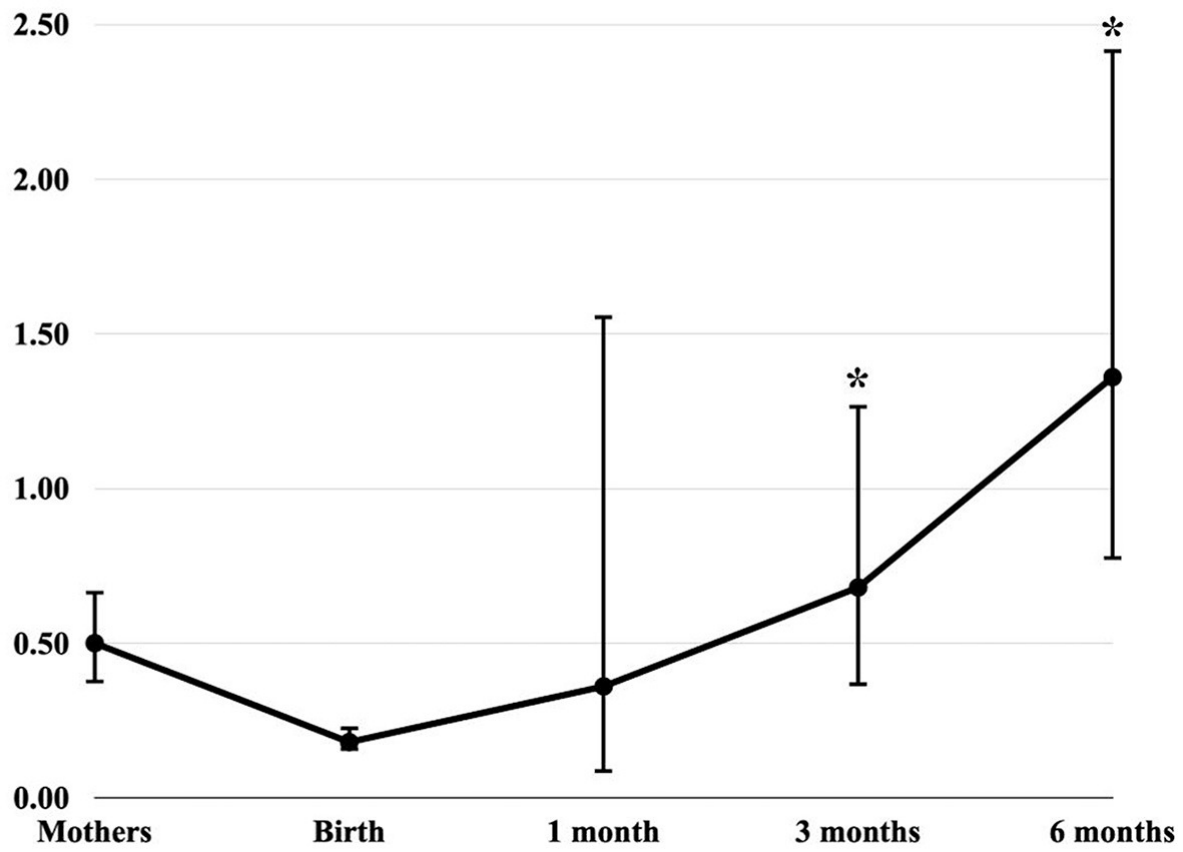
Creatinine-adjusted levels of 3-PBA in pregnant women and their newborns. Creatinine-adjusted GM and 95% CI of 3-PBA levels (ng/mg). Mothers (pregnant women in the 3rd trimester of gestation): 0.50 (0.37–0.67); Birth: 0.18 (0.15–0.23); 1 month: 0.36 (0.08–1.56); 3 months: 0.68 (0.36–1.27); and 6 months: 1.36 (0.77–2.42). * Significantly different from birth levels at *p* < 0.05. Detection rates: Mothers = 47.2% (*n* = 67); Birth = 10.2% (*n* = 8); 1 month = 9.4% (*n* = 5); 3 months = 20.9% (*n* = 14); and 6 months 20.7% (*n* = 12).

Figure 1 shows the creatinine-adjusted GM and 95% CI of 3-PBA levels (ng/mg) in pregnant women and their children, according to the follow-up ages. The metabolite 3-PBA was detected in 47.2% of the pregnant women, while 4-FPBA was found in 10.6% of them. In children's urine samples, only 3-PBA was detected. The detection rate of 3-PBA at birth was 10.2%, and 9.4, 20.9, and 20.7% in the 1^st^, 3^rd^, and 6^th^ months of life, respectively. The GM and 95% CI of 3-PBA levels were 0.50 (0.37–0.67) ng/mg for the pregnant women, 0.18 (0.15–0.23) ng/mg at birth, 0.36 (0.08–1.56) ng/mg at 1-month-old, 0.68 (0.36–1.27) ng/mg at 3-month-old, and 1.36 (0.77–2.42) ng/mg at 6-month-old. The Kruskal-Wallis test suggests that the difference in 3-PBA levels across children's age groups is significant (*p* = 0.001). *Post hoc* analysis using Dunn's test indicates that urinary concentrations of 3-PBA in 3- and 6-month-old children are different from those at birth (*p* < 0.05).

## Discussion

This research analyzed the levels of 3-PBA in the urine of pregnant women and their children. Moreover, 10% of children's samples had detectable levels of 3-PBA when they were born, which increased to 21% in the third and 6^th^ months of their lives. The presence of pyrethroid metabolites in the first urination of infants suggests that they were exposed to them through their mothers. Additionally, the levels of urinary pyrethroids observed in the follow-up months suggest that infants were exposed to them through breast milk, food consumption, and the use of pyrethroid-based insecticides indoors.

In pregnant women, 3-PBA was more frequently detected than 4-FPBA as it is a metabolite of commonly used pyrethroids in Brazil such as cypermethrin, cyhalothrin, deltamethrin esfenvalerate, and permethrin, while 4-FPBA is a metabolite derived from cyfluthrin. Although the detection rate of 3-PBA was lower in our study, its geometric mean was higher compared to the pregnant women in Porto, Portugal, and Tokyo, Japan (26, 27). However, in a study conducted in China's agricultural area, Qi and colleagues (16) detected 3-PBA in 94.1% of urine samples from pregnant women, with an average level of 1.53 μg/g, which is higher than we observed. This suggests that proximity to agricultural areas may increase exposure to pyrethroids. Our results converge with those observed in a survey of the adult population living in the city of Rio de Janeiro, conducted by Rosa (28) in 2015. In Rosa's study, 156 (33%) participants showed detectable levels of 3-PBA, which is lower than the 47% detected in our study. Rosa also reported that 3-PBA median and interquartile range levels were 0.84 (0.35–1.60) ng/g of creatine, while in our study these parameters were 0.50 (0.37–0.67) ng/g of creatine.

Few studies have looked at 3-PBA urinary levels in children at birth and in the early stages of life. In rural Yunnan, China (29), for example, the detection rate of 3-PBA in 6- to 8-month-old infants was 65.9%, with 0.25 g/g median levels in urine. A creatinine-adjusted average level of 3-PBA was 2.25 g/g in Ichinomiya and Nagoya, both in Japan, and was detected in 98% of the 1,075 urine samples from 16–23-month-old enrolled children (30). The creatinine-adjusted geometric mean of urinary 3-PBA and its detection rates were lower in our study than in the previous studies conducted in China and Japan. Other studies on urinary 3-PBA concentrations in infants and children have been conducted, but the results have not been adjusted for urinary creatinine levels. For example, a study conducted in the United States discovered 0.07 g/L of urinary 3-PBA in 11% of 14–30 month-old children (31). In Jiangsu, China, geometric means of 3-PBA at 0.44 ng/ml of urine were found in 83.9% of children under the age of 1 year (29).

In our study, the detection rate of 3-PBA in newborns at birth was low. However, the prevalence of samples with concentrations of 3-PBA above the LOD increased on 3rd and 6th-month follow-ups. This finding is concerning because newborns and neonates are especially vulnerable to the effects of pollutants due to their immature nervous and immune systems (30, 31). It's possible that insecticide use in households decreases during the first few months of a child's life due to more attentive care. Furthermore, due to the widespread use of pyrethroids in households, fetuses may be exposed through the placenta, and children can be exposed during breastfeeding (22, 32). Additionally, infants and children may also be exposed to these substances through household dust and clothes (33).

Breast milk is considered the most beneficial source of nutrition for babies, aiding in their growth and development. Despite lactation being a route of pollutant excretion and consequently exposure to infants, until now, there is no evidence to discourage breastfeeding (34). However, it is important to understand how pollutants are excreted at different times during lactation. Studies suggest that some pollutants are excreted more in colostrum than in mature milk (35, 36). While the number of children with detectable levels of 3-PBA in their urine during the first 6 months of life and after transitioning from breastfeeding to formulae and baby food was too small for statistical comparison, the observed increase highlights the possible role that diet may play in a baby's exposure to pyrethroids. This hypothesis should be explored in further studies.

Due to the small sample size and convenience sampling method used for the women's recruitment, the data may not accurately represent the entire population of pregnant women and children in Rio de Janeiro. Furthermore, a larger sample size would be needed to conduct a multivariate analysis or adjust for covariates to identify 3-PBA predictors, and the study only analyzed a limited selection of pyrethroid metabolites. These issues are expected to be addressed in the main cohort study.

This is one of the few studies that investigated the pyrethroid concentrations in the urine of newborns and children in their 1^st^ year of life. We found that approximately half of the pregnant women had detectable levels of 3-PBA. Our results also showed that, despite low, children's exposure to pyrethroids significantly increased after birth, suggesting that children's exposure to pyrethroids starts early in life.

## Data availability statement

The raw data supporting the conclusions of this article will be made available by the authors, without undue reservation.

## Ethics statement

The studies involving humans were approved by Ethics Committee of the Maternity Hospital, Federal University of Rio de Janeiro (protocol 2.092.440). The studies were conducted in accordance with the local legislation and institutional requirements. Written informed consent for participation in this study was provided by the participants' legal guardians/next of kin.

## Author contributions

AFM: Formal analysis, Investigation, Writing – original draft. AS: Conceptualization, Data curation, Formal analysis, Investigation, Methodology, Writing – original draft, Writing – review & editing. JM: Writing – review & editing, Writing – original draft. VC: Conceptualization, Supervision, Writing – review & editing. CA: Conceptualization, Data curation, Funding acquisition, Investigation, Methodology, Project administration, Supervision, Writing – review & editing. AR: Writing – review & editing, Formal analysis, Methodology. PV: Writing – review & editing. AM: Conceptualization, Data curation, Funding acquisition, Methodology, Supervision, Writing – review & editing.
